# Potent USP10/13 antagonist spautin‐1 suppresses melanoma growth via ROS‐mediated DNA damage and exhibits synergy with cisplatin

**DOI:** 10.1111/jcmm.15093

**Published:** 2020-03-04

**Authors:** Jia Guo, JiangLing Zhang, Long Liang, Nian Liu, Min Qi, Shuang Zhao, Juan Su, Jing Liu, Cong Peng, Xiang Chen, Hong Liu

**Affiliations:** ^1^ Department of Dermatology Xiangya Hospital Central South University Changsha China; ^2^ Hunan Key Laboratory of Skin Cancer and Psoriasis Changsha China; ^3^ Hunan Engineering Research Center of Skin Health and Disease Changsha China; ^4^ Molecular Biology Research Center and Center for Medical Genetics Central South University Changsha China; ^5^ Department of Plastic and Cosmetic Surgery Xiangya Hospital Central South University Changsha China; ^6^ Xiangya Clinical Research Center for Cancer Immunotherapy Central South University Changsha China; ^7^ Research Center of Molecular Metabolomics Xiangya Hospital Central South University Changsha China

**Keywords:** combination therapy, DNA damage, malignant melanoma, ROS, spautin‐1

## Abstract

Malignant melanoma is one of the most invasive tumours. However, effective therapeutic strategies are limited, and overall survival rates remain low. By utilizing transcriptomic profiling, tissue array and molecular biology, we revealed that two key ubiquitin‐specific proteases (USPs), ubiquitin‐specific peptidase10 (USP10) and ubiquitin‐specific peptidase10 (USP13), were significantly elevated in melanoma at the mRNA and protein levels. Spautin‐1 has been reported as a USP10 and USP13 antagonist, and we demonstrated that spautin‐1 has potent anti‐tumour effects as reflected by MTS and the colony formation assays in various melanoma cell lines without cytotoxic effects in HaCaT and JB6 cell lines. Mechanistically, we identified apoptosis and ROS‐mediated DNA damage as critical mechanisms underlying the spautin‐1‐mediated anti‐tumour effect by utilizing transcriptomics, qRT‐PCR validation, flow cytometry, Western blotting and immunofluorescence staining. Importantly, by screening spautin‐1 with targeted or chemotherapeutic drugs, we showed that spautin‐1 exhibited synergy with cisplatin in the treatment of melanoma. Pre‐clinically, we demonstrated that spautin‐1 significantly attenuated tumour growth in a cell line‐derived xenograft mouse model, and its anti‐tumour effect was further enhanced by cotreatment with cisplatin. Taken together, our study revealed a novel molecular mechanism of spautin‐1 effecting in melanoma and identified a potential therapeutic strategy in treatment of melanoma patients.

## 1 INTRODUCTION

Malignant melanoma, a solid tumour produced by the malignant transformation of melanocytes from the skin and other organs, has an increasing incidence and a poor prognosis.^1^ Compared with other tumours originating from the epidermis and accessory organs, the cutaneous form of the disease is common in the Western world and is late onset with no obvious early symptoms, which causes the three‐quarters of the deaths related to skin cancer.^2^ The early stage of melanoma has a good prognosis after sufficient surgical treatment. However, the advanced stage of melanoma is still a therapeutic challenge despite the development of multiple new therapeutic methods. Although chemotherapeutics or targeted therapeutics alter tumour growth, in most cases, the effect is not long‐lasting resulting in drug resistance that causes treatment failure and impacts the survival of patients.^3^, ^4^ New immunotherapies have been recently approved to induce long‐lasting responses in patients with metastatic cancers, even so, resistance and poor response rates still persist.^5^ As melanoma eventually becomes resistant to these novel therapies, the development of more effective approaches is required.

Ubiquitination is a post‐translational protein modification that is regulated by a series of ubiquitination‐associated enzymes, including the ubiquitin‐activating enzyme (E1), the ubiquitin‐conjugating enzyme (E2), the ubiquitin ligase (E3) and deubiquitinases (DUBs).^6^ Ubiquitination spans wide‐spectrum functions in the cell that include cell death, DNA damage repair and protein degradation.^7^ Thus, dysregulation of ubiquitination leads to an imbalance between promoting and suppressing pathways of tumours. Recently, accumulative evidence has established the critical role of ubiquitination in cancer pathogenesis and has therefore revealed a potential therapeutic role in various cancers. In particular, the advent of some new inhibitors revealed suppressive effect in melanoma.^8^, ^9^ Ubiquitin‐specific peptidase10 (USP10) and ubiquitin‐specific peptidase 13 (USP13) both belong to USP family, the largest subfamily of DUB.^10^ USP10 and USP13 are involved in a number of biological processes and impact the development of various tumours by stabilizing several proteins. USP13 and USP10 usually function as functional partners to regulate the stability of several proteins, such as p53.^11^, ^12^ Whether targeting these two molecules can inhibit the melanoma or not is still unclear.

In this study, we employed a potent USP10/13 deubiquitinating activity antagonist, spautin‐1, to investigate the effect of targeting USP10/USP13 as an anti‐melanoma treatment and found that melanoma cell proliferation was significantly suppressed by spautin‐1‐induced ROS‐mediated DNA damage. Besides, spautin‐1 treatment could combine with cisplatin, a well‐known chemotherapeutic drug, which was shown to enhance the anticancer effect of cisplatin both in vivo and in vitro.

## 2 MATERIALS AND METHODS

### 2.1 NCBI GEO data analysis

To identify the expression of USP10 and USP13 in malignant melanoma, data were obtained from the GEO database. The raw data set http://www.ncbi.nlm.nih.gov/geo/query/acc.cgi?acc=GSE3189 was downloaded, which provided the USP10 and USP13 miRNA profiles including 7 normal skin samples, 18 naevus samples and 45 melanoma samples. These data were analysed based on the GPL96 platform.

### 2.2 Cell lines and culture

The human malignant melanoma cell lines (A375 and SK‐Mel‐28), the human keratinocyte HaCaT and the mouse epidermis‐derived JB6 Cl 41‐5a cells were purchased from the American Type Culture Collection (ATCC). All cells were grown in Dulbecco's modified Eagle's medium (BI) supplemented with 10% foetal bovine serum (BI) at 37°C in a 5% CO_2_ humidified incubator.

### 2.3 MTS assay

Cells (2 × 10^3^ per well) were seeded in a 96‐well plate and allowed to adhere overnight in the culture medium containing 10% foetal bovine serum. Then, the cells were treated with various concentration of spautin‐1 (control, 2.5, 5, 10, 20 μmol/L) for 24, 48 and 72 hours or a combination of spautin‐1 with cisplatin (2, 4, 6, 8 μmol/L), vemurafenib (0.05, 0.1, 0.5, 1 μmol/L) or trametinib (0.5, 1, 2, 5 nmol/L) 72 hours, respectively. The effect of spautin‐1 or a combination of spautin‐1 and cisplatin on cell viability was tested using the Non‐radioactive Cell Proliferation Assay (3‐(4,5‐dimethylthiazol‐2‐yl)‐5‐(3‐carboxymethoxyphenyl)‐2‐(4‐sulfophenyl)‐2H‐tetrazolium, MTS) (Promega) according to the manufacturer's instructions.

### 2.4 Colony formation assay

Cells were seeded into 24‐well plates (0.5‐1 × 10^3^ cells per well) and incubated overnight. Then, the cells were exposed to various concentration of spautin‐1 (2.5, 10 μmol/L) or vehicle control (DMSO). After 48 hours, the drug‐containing medium was replaced with complete growth medium for two weeks until the visible colony formation. During the process, the medium was refreshed every three days. Finally, the cells were washed with PBS, fixed with 4% paraformaldehyde (Servicebio) and stained with 0.5% crystal violet (DingGuo). At the end‐point, the images were captured.

### 2.5 Cell transfection

Negative control small interfering siRNA (si‐NC), USP10 small interfering RNA (si‐USP10) and USP13 small interfering RNA (si‐USP13) were constructed by Gene Pharma. For transfection experiments, A375 and Sk‐Mel‐28 cells (2 × 10^5^ per well) were seeded in a 6‐well plate to allow attachment and culture for 24 hours, grown to 70%‐80% confluence the day of transfection. The cells were cotransfected with different oligonucleotides using TurboFect (Thermo Scientific) following the manufacturer's protocol. Cells were used to subsequent experiments after transfection for 48 hours.

### 2.6 Cell apoptosis and cell cycle assay

Apoptosis and cell cycle distribution were detected by flow cytometer. For the cell cycle assays, cells treated with various concentrations of spautin‐1 (0, 2.5, 10 μmol/L) were trypsinized and washed with cold PBS. Then, the collected cells were suspended in 75% ethanol at 4°C overnight. The next day, the cells were stained with 0.5 mL of propidium iodide (PI) staining (Beyotime Biotechnology) according to the manufacturer's instructions. The data were analysed using ModFit software. For the cell apoptosis, the treated cells were digested by trypsin solution without EDTA (Beyotime Biotechnology), washed with PBS, and incubated with 5 µL annexin V staining for minutes at room temperature, and then stained with 10 µL of propidium iodide (BD Biosciences) before being detected. The samples were run on a DxP cytometer (Cytek), and the data were analysed by FlowJo 10 software.

### 2.7 Western blotting

Cells were harvested in RIPA Lysis Buffer (DingGuo) with a protease inhibitor and phosphatase inhibitors (Selleck), and protein concentration was determined using a BCA assay kit (Beyotime). Protein samples (30 μg) were subjected to 8%‐12% SDS‐polyacrylamide gel electrophoresis (SDS‐PAGE) and then transferred onto polyvinylidene fluoride membranes (Millipore). The membranes were blocked with 5% non‐fat milk or 5% bovine serum albumin (BSA) for one hour at room temperature and then incubated with following primary and secondary antibodies: USP10 (1:1000, CST), USP13 (1:1000, Proteintech Group), PARP (1:1000, CST), BAX (1:1000, CST), BCL2 (1:1000, CST), CDC2 (1:1000, Proteintech Group), CyclinB1 (1:1000, Santa), γ‐H2AX (1:1000, CST), p‐ATM (1:1000, CST), p‐ATR (1:1000, CST), ATM (1:1000, Proteintech Group), ATR (1:1000, Proteintech Group) or GAPDH (1:3000, Proteintech Group). The blots were detected and analysed using a gel image analysis system (LI‐COR).

### 2.8 Immunofluorescence assay

Cells (2 × 10^5^ per well) were seeded on coverslips in a 6‐well plate to allow attachment and then exposed to spautin‐1 (10 μmol/L) for 0, 24 or 48 hours. Cells were fixed in 4% paraformaldehyde for 20 minutes and permeabilized with 0.5% Triton X‐100 for 1 hour. After blocking with 5% bovine serum albumin (BSA) for 1 hour, cells were incubated with γ‐H2AX (1:100, CST) and secondary antibody (Invitrogen), and then stained with DAPI (Servicebio) to visualize nuclear DNA. The images were captured by confocal laser scanning microscope (TCS‐SP8; Leica Microsystems) for Alexa Fluor 594 and DAPI.

### 2.9 Mitochondrial membrane potential detection

Cells (2 × 10^5^ per well) were seeded on coverslips in a 6‐well plate to allow attachment and then exposed to spautin‐1 (10 μmol/L) for 0, 6, 12 or 24 hours. Then, the cells were incubated with 1 mL of JC‐1 working solution for 30 minutes according to the manufacturer's instruction (Beyotime Biotechnology). After being washed with JC‐1 staining buffer twice, the cells were observed and captured by fluorescence microscopy (Ts2R, Nikon).

### 2.10 Measurement of ROS generation

The effect of spautin‐1 on intracellular ROS generation was measured using a DCFH‐DA reactive oxygen species ROS fluorescence probe (Solarbio). Cells (3 × 10^5^ per well) were seeded in a 6‐well plate to allow attachment and then exposed to spautin‐1 for 0, 3, 6 or 12 hours. Then, the cells were incubated with DCFH‐DA for 30 minutes according to the manufacturer's instruction. The intracellular ROS generation was measured by a DxP Athena cytometer (Cytek), and the data were analysed by the FlowJo10 software.

### 2.11 Statistical analysis of drug interaction

The intensity and properties of the interaction of spautin‐1 and cisplatin could be quantitatively determined by the combination index (CI) of the Chou‐Talalay method using the Calcusyn version 2.0. A CI value >1 was antagonistic, equal to 1 was considered to be additive and <1 was synergistic.

### 2.12 Cell‐derived xenograft in vivo melanoma mouse model

A375 cells (5 × 10^6^/100 mL) were subcutaneously injected into the right flank of 4‐ to‐ 6‐week‐old BALB/C nude mice. When the tumour volume reached 50‐100 mm^3^, the tumour‐bearing nude mice were randomly divided into four groups and treated with vehicle (Control), spautin‐1 (40 mg/kg every other day), cisplatin (3 mg/kg once a week) or a combination of both drugs for 2 weeks. The length, width and height of the tumour and the weight of the tumour‐bearing mice were measured every 2 days. The tumour volumes (mm^3^) were calculated by the formula (length × width ×height × π/6). At the end of the experiment, the tumour‐bearing mice were sacrificed, and the xenografts were removed for histology and further analysis.

### 2.13 Immunohistochemistry

The tumour tissue was made into sections. After a series of dewaxing and gradient alcohol dehydration, antigen repair was performed in a microwave. The slices were cooled to room temperature and washed 3 times with PBS. An immunohistochemical pen was used to draw a circle, and peroxidase was blocked for 10 minutes. The primary antibodies against Ki67 (1:400, Abcam) and γ‐H2AX (1:200, CST) were applied to the slides which were incubated in a humidified chamber at 4℃ overnight. The next day, the slides were incubated with the secondary antibody for 30 minutes, and then the reaction was visualized by DAB staining. After that, the slides were counterstained with haematoxylin. Finally, after a series of dehydrations, the sections were sealed with cover glass and neutral resin.

### 2.14 RNA‐sequencing analysis

RNA‐seq was completed on the BGISEQ‐500 platform (BGI). After the quality control was adjusted, the quantitative analysis of the gene was carried out based on the analysis of the gene expression level (principal component, correlation, differential gene screening, etc). The differentially expressed genes between samples were analysed by Gene Ontology (GO), KEGG pathway, enrichment cluster, protein interaction network, and other in‐depth analyses according to BGI’s instructions.

### 2.15 Quantitative real‐time PCR

Total RNA was extracted from cells using TRIzol reagent (Bioteke Corporation), then reverse transcribed into cDNA using HiScript II Q RT SuperMix for qPCR (Vazyme) according to the manufacturer's instructions. Then, 40 cycles of quantitative reverse‐transcription PCR (qRT‐PCR) were conducted in 96‐well plates using SYBR Green qPCR mixture (CWBIO) on the QuantStudio3 Real‐Time PCR System. The fold change of gene expression was calculated by 2^−(ΔCtexperimental group − ΔCtcontrol group)^. The sequence of primers is listed in Table S1.

### 2.16 Statistical analysis methods

Data are presented as the mean ± SEM. Statistical analysis was performed using GraphPad Prism software (version 6.01). Differences between the means of 2 groups were compared by Student's *t* test, and the differences of multiple groups were compared by one‐way ANOVA, followed by a Tukey multiple‐comparisons test. *P* < .05 was considered significant.

## 3 RESULTS

### 3.1 USP10 and USP13 mRNA and protein levels were elevated in melanoma patient samples and various human melanoma cell lines

**Figure 1:**
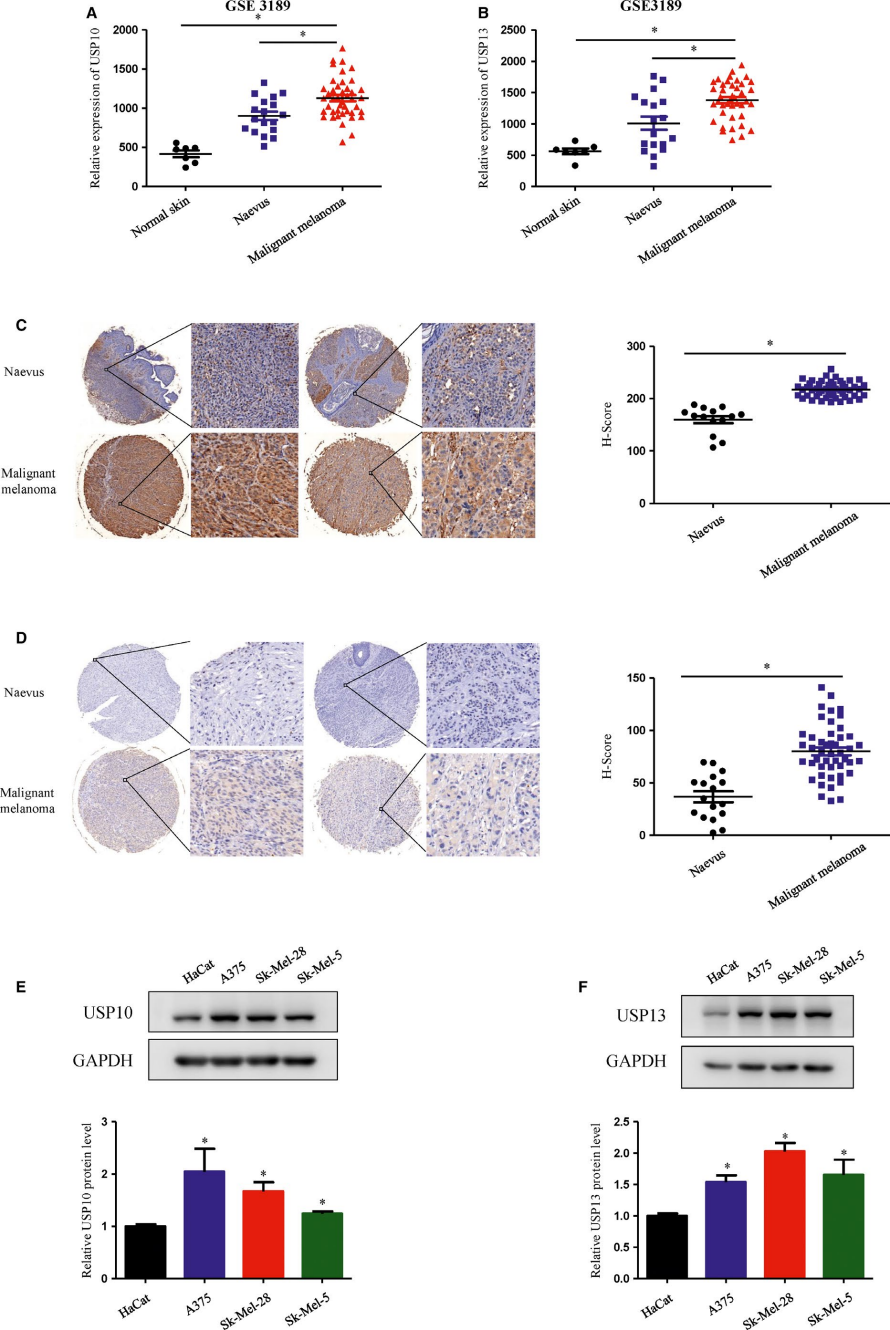
USP10 and USP13 mRNA and protein levels were elevated in tumour tissue of melanoma patients and in melanoma human cell lines. A and B, USP10 (A) and USP13 (B) expression in normal skin (n = 7), naevus (n = 18) and malignant melanoma (n = 45) in the http://www.ncbi.nlm.nih.gov/geo/query/acc.cgi?acc=GSE3189 data sets. A, USP10 mRNA expression in the http://www.ncbi.nlm.nih.gov/geo/query/acc.cgi?acc=GSE3189 data sets. Significant differences were evaluated using Student's *t* test. *P < .05 vs malignant melanoma. B, USP13 mRNA expression in the http://www.ncbi.nlm.nih.gov/geo/query/acc.cgi?acc=GSE3189 data sets. Significant differences were evaluated using Student's *t* test. *P < .05 vs malignant melanoma. C and D, Immunohistochemical staining of USP10 (C) and USP13 (D) in normal naevi tissues and malignant melanoma. Right panel: quantification of USP10 and USP13 in naevi and melanoma tissues. Significant differences were evaluated using Student's *t* test. *P < .05 vs naevus. E and F, The protein level of USP10 (E) and USP13 (F) in the human skin keratinocytes cell line (HaCat) and different melanoma cell lines were measured by Western blot (upper panel). Semi‐quantitative analysis of USP10 and USP13 proteins expression compared with GAPDH. (Lower panel) (mean values ± SEM, n = 3) Significant differences were evaluated using Student's *t* test. *P < .05 vs HaCat

USP10 and USP13 expression was significantly up‐regulated in the melanoma tissues compared to naevus or normal skin tissue according to the http://www.ncbi.nlm.nih.gov/geo/query/acc.cgi?acc=GSE3189 data sets (Figure 1A,B). To assess the role of USP10 and USP13 in melanoma progression, we applied melanoma tissue microarray and immunohistochemistry to detect their expression. As shown in Figure 1C,D, USP10 and USP13 were highly expressed in malignant melanoma specimens compared to the naevus tissue. We have checked the expression of USP10 and USP13 in different melanoma cell lines and human skin keratinocytes cell line (HaCaT). We found that there were higher USP10 and USP13 expression in melanoma cell lines compared with control cells. In particular, their expression was relatively higher in A375 and SK‐Mel‐28 melanoma cell lines compared to Sk‐Mel‐05 melanoma cell line (Figure 1E,F). All the results suggested that USP10 and USP13 might play detrimental roles in melanoma.

### 3.2 Spautin‐1, a dual USP10 and USP13 antagonist, inhibits melanoma cell growth in vitro

**Figure 2:**
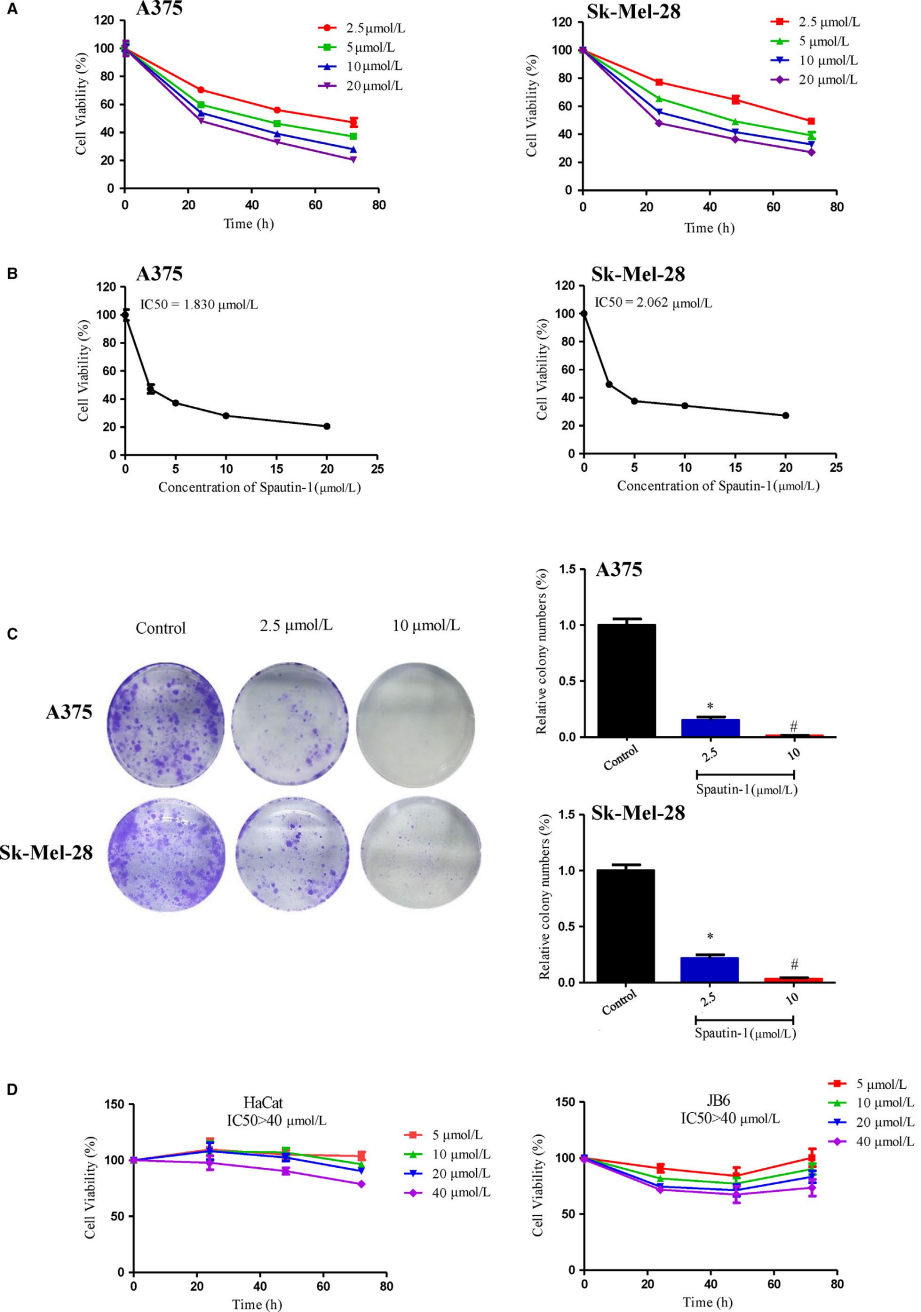
Spautin‐1 significantly attenuated proliferation of melanoma cells in a time‐ and dose‐dependent manner. A, Viability analysis after the treatment of A375 and Sk‐Mel‐28 cells with different concentration of spautin‐1 (up to 20 µmol/L) for 0, 24, 48 and 72 h. (Mean values ± SEM, n = 6). B, The IC50 values of spautin‐1 in A375 and Sk‐Mel‐28 were automatically calculated by GraphPad Prism software. C, spautin‐1 inhibited A375 and Sk‐Mel‐28 cell colony formation. The number of colonies normalized to the corresponding untreated cells formed colonies. (Mean values ± SEM, n = 3) Significant differences were evaluated using a one‐way ANOVA. *P < .05 vs control group, ^#^ *P* < .05 vs 2.5 µmol/L. D, Non‐tumorigenic cell lines (HaCaT and JB6) were treated with various concentration of spautin‐1 for 72 h as indicated, and cell viability in the presence of HaCat and JB6 was measured by MTS as described in the Section 2 and analysed by GraphPad Prism software (mean values ± SEM, n = 6)

Spautin‐1, as a potential USP10/13 antagonist, was reported to play an anti‐tumour effect in various cancers, such as chronic myeloid leukaemia, ovarian cancer and lung cancer.^13^ In this study, we investigated the role of spautin‐1 in malignant melanoma. First, an MTS assay was performed to determine the effects of spautin‐1 on the viability of different melanoma cell lines. As shown in Figure 2A, spautin‐1 markedly decreased the proliferation of A375 and SK‐Mel‐28 in a dose‐ and time‐dependent manner. The 72‐hour IC50 values of spautin‐1 for A375 and SK‐Mel‐28 were 1.830 and 2.062 μmol/L, respectively (Figure 2B). Similar results were observed by knock‐down of USP10/USP13 in A375 and Sk‐Mel‐28 (Figure S2B). Next, the colony formation assays were used to further study the effect of spautin‐1 on melanoma cell lines. The results indicated that the formation of cell colonies was suppressed by spautin‐1 in a dosage‐dependent manner (Figure 2C). Moreover, we investigated whether spautin‐1 treatment had selective effects on melanoma cells. The results show the 72‐hour IC50 value of for two normal cell lines, HaCaT and JB6, was much greater than the efficacious dose for A375 and SK‐Mel‐28 (Figure 2D), which indicated that spautin‐1 inhibited the proliferation of melanoma cell lines without significant effects in normal cells. Spautin‐1 did not cause obvious normal cell death either (Figure S1A). The overall results indicated that spautin‐1 had a potent anti‐proliferative effect on melanoma cells without an obvious cytotoxic effect on normal cells.

### 3.3 Transcriptomics coupled with qRT‐PCR validation identified a potential mechanism of spautin‐1‐mediated anti‐tumour growth effects

**Figure 3:**
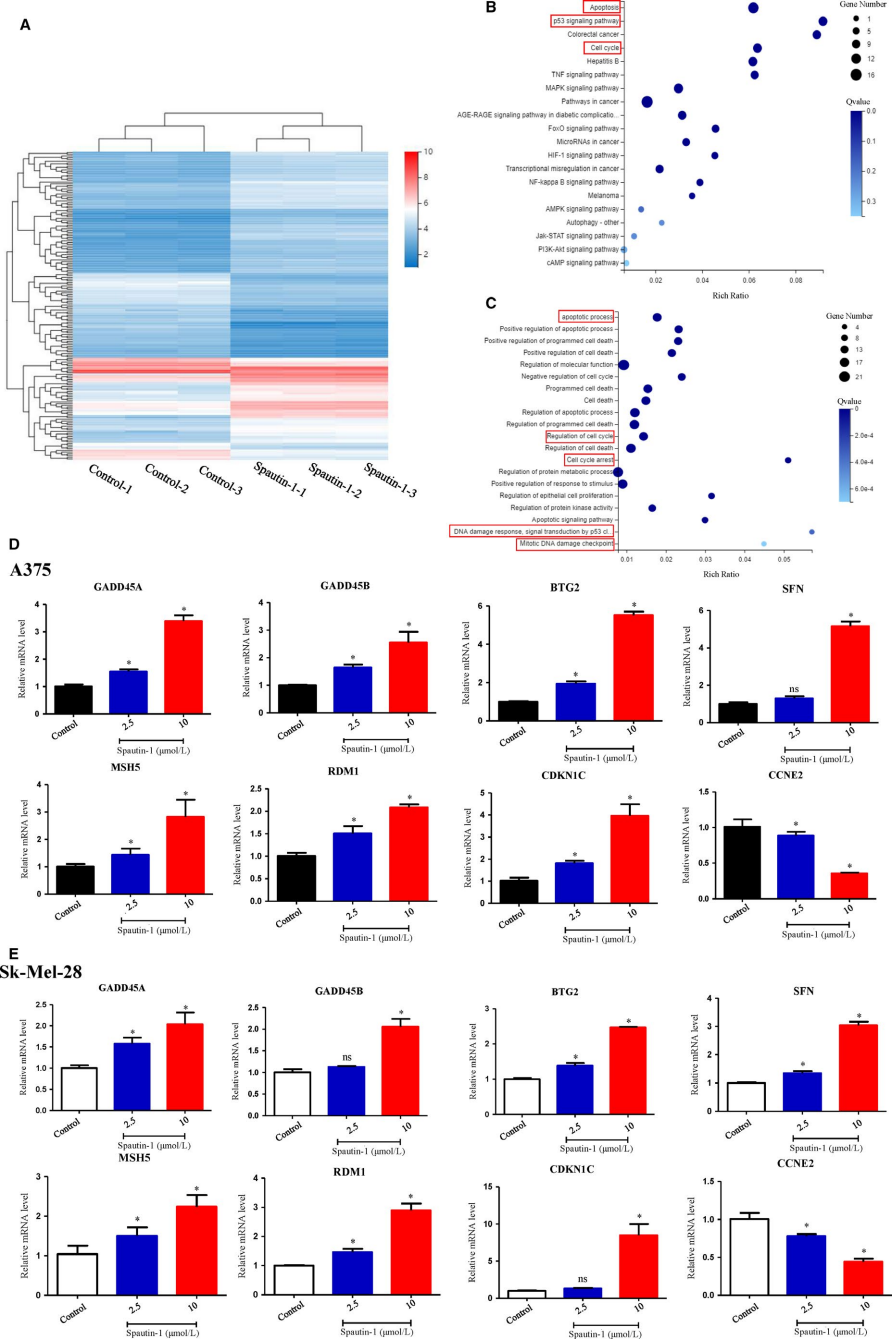
Transcriptomics coupled with qRT‐PCR identified apoptosis and DNA damage as potential mechanisms underlying spautin‐1‐mediated anti‐tumour effect. A, Sk‐Mel‐28 cells were treated with 10 µmol/L Spautin‐1 for 48 h. Cluster analysis of the genes expressed in each comparison group, displaying with a heat map. B, The pathways correlate with the differential expression genes was analysed by enriched KEGG pathway. The enriched bubble chart shows the relative top 20 enriched pathways. C, Gene Ontology analysis of the relative differential expression genes. Only top 20 enriched pathways are shown in the bubble chart. D and E, A375 (D) and Sk‐Mel‐28 (E) were treated with various dosages of spautin‐1 for 48 h. The key differential expression genes related to cell cycle and DNA damage were validated by qRT‐PCR. (Mean values ± SEM, n = 3) Significant differences were evaluated using a one‐way ANOVA. *P < .05 vs control

To identify the molecular mechanism of the spautin‐1‐mediated inhibitory effect on melanoma cell growth, we analysed the global transcriptomic alteration of melanoma cells after treatment with spautin‐1. Bioinformatic analysis identified that 699 genes were up‐regulated, and 356 genes were down‐regulated after spautin‐1 treatment for 48 hours (Figure 3A). Furthermore, the relative differentially expressed genes were analysed by the Kyoto Encyclopedia of Genes and Genomes (KEGG) pathway and Gene Ontology (GO) analysis. The top 20 enriched pathways related to cell proliferation are shown in Figure 3B,C. It is suggested that pathways including cell cycle, apoptosis, PI3K‐Akt and DNA damage‐related pathway were ranked among the top pathways and that those pathways may play an important role in the mechanism of spautin‐1 inhibition of the growth of melanoma. Then, qRT‐PCR was utilized to further validate the transcriptomic results by quantifying several key genes related to cell cycle and DNA damage, including growth arrest and DNA damage‐inducible 45 alpha (GADD45A), growth arrest and DNA damage‐inducible 45 beta (GADD45B), STRATIFIN (SFN), RAD52 motif containing 1 (RDM1), mutS homologue 5 (MSH5), cyclin dependent kinase inhibitor 1C (CDKN1C) and BTG anti‐proliferation factor 2 (BTG2) (Figure 3D,E).

### 3.4 Spautin‐1 induced G2/M cell cycle arrest and cell apoptosis via up‐regulation of ROS‐mediated DNA damage in melanoma cell lines in vitro

**Figure 4:**
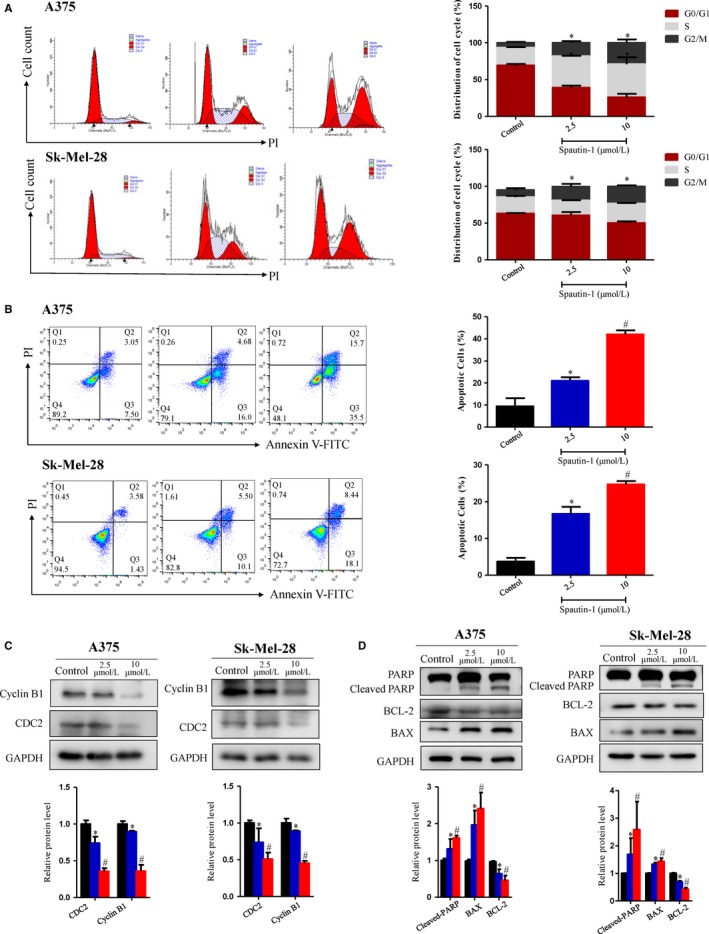
Flow cytometry and Western blot validated that spautin‐1 induce G2/M cell cycle arrest and increased cell apoptosis in melanoma cell lines. A, Representative data (Left) and quantitative analysis (Right) of the cell cycle distribution of spautin‐1‐treated cells. A375 and Sk‐Mel‐28 cells were treated with spautin‐1 at the indicated concentrations for 48 h. Cell cycle profile was detected using flow cytometry, and the data were analysed by ModFit software. (Mean values ± SEM, n = 3) Significant differences were evaluated using a one‐way ANOVA. *P < .05 vs control. B, (Left) Representative data of apoptosis of spautin‐1‐treated cells. A375 and Sk‐Mel‐28 cells were disposed with different dosages of spautin‐1 for 48 h and then cells were stained with annexin V and PI to measure the percentage of apoptotic cells. (Right) The bar graphs showed the percentage of apoptotic cells, early apoptosis (annexin V positive, PI negative) and late apoptosis (annexin V positive, PI positive). (Mean values ± SEM, n = 3) Significant differences were evaluated using a one‐way ANOVA. *P < .05 vs control group, ^#^ *P* < .05 vs 2.5 µmol/L. C, Cells were treated with control (DMSO), spautin‐1 (2.5 or 10 μmol/L) for 48 h. Western blot was used to analysis the expression of G2/M phase arrest‐associated proteins, including CDC2 and Cyclin B1. GAPDH was used as an internal control (upper panel). Semi‐quantitative analysis of the proteins expression compared with GAPDH. (Lower panel) (Mean values ± SD, n = 3) Significant differences were evaluated using a one‐way ANOVA. *P < .05 vs control group, ^#^ *P* < .05 vs 2.5 µmol/L. D, A375 and Sk‐Mel‐28 cells were treated with control (DMSO), spautin‐1 (2.5 or 10 μmol/L) for 48 h. Western blot assay was then implied to detect the indicated protein level. GAPDH was used as an internal control (upper panel). Semi‐quantitative analysis of cleaved‐PARP proteins expression compared with PARP, BAX and BCL2 proteins expression compared with GAPDH. (Lower panel) (Mean values ± SEM, n = 3) Significant differences were evaluated using a one‐way ANOVA. *P < .05 vs control group, ^#^ *P* < .05 vs 2.5 µmol/L

To test whether spautin‐1‐mediated anti‐proliferation in melanoma is via regulating cell cycle distribution and cell apoptosis, we analysed the cell cycle distribution and cell apoptosis of A375 and SK‐Mel‐28 with or without spautin‐1 treatment. Intriguingly, we found that spautin‐1 could strongly induce G2/M cell cycle arrest and increase cell apoptosis in a dose‐dependent manner in multiple melanoma cell lines. Spautin‐1 shifted the percentage of cells in G2/M phase from 6.24% and 9.63% in the vehicle control to 18.35% and 18.56% at 2.5 μmol/L and 29.05% and 23.68% at 10.0 μmol/L of treatment with spautin‐1 for A375 and SK‐Mel‐28 cells, respectively (Figure 4A). To further clarify the effects of spautin‐1‐induced G2/M phase arrest, Western blotting was performed to examine the expression of G2/M phase arrest‐associated proteins during spautin‐1 treatment. As shown in Figure 4C, spautin‐1 treatment markedly decreased the expression of the Cyclin B1 and CDC2, two important proteins involved in G2/M phase transition. Furthermore, we quantified the percentage of spautin‐1‐induced apoptotic cells in melanoma cell lines. At the concentration of 2.5 μmol/L, spautin‐1 induced 21.03% and 16.68% apoptosis in A375 and Sk‐Mel‐28, respectively. At 10 μmol/L, apoptosis increased to 42.03% and 24.72% in A375 and Sk‐Mel‐28 (Figure 4B). Knock‐down of USP10/USP13 increased cellular apoptotic rate approximately 2‐3 folds in A375 or Sk‐Mel‐28 human melanoma cells (Figure S2C). In contrast to the above cancer cell lines analysed, HaCaT and JB6 cells treated with spautin‐1 did not show significant apoptosis (Figure S1A). In addition, the expression of cleaved‐PARP, BAX and BCL‐2, which are all apoptosis‐related proteins, was further detected. After treatment with spautin‐1 in A375 and SK‐Mel‐28, the protein levels of cleaved‐PARP and BAX were up‐regulated, while the BCL‐2, an anti‐apoptotic protein, was decreased compared with the DMSO group (Figure 4D). These results indicate that spautin‐1 inhibited the proliferation of malignant melanoma cells by inducing G2/M cell cycle arrest and increasing cell apoptosis in vitro.

**Figure 5:**
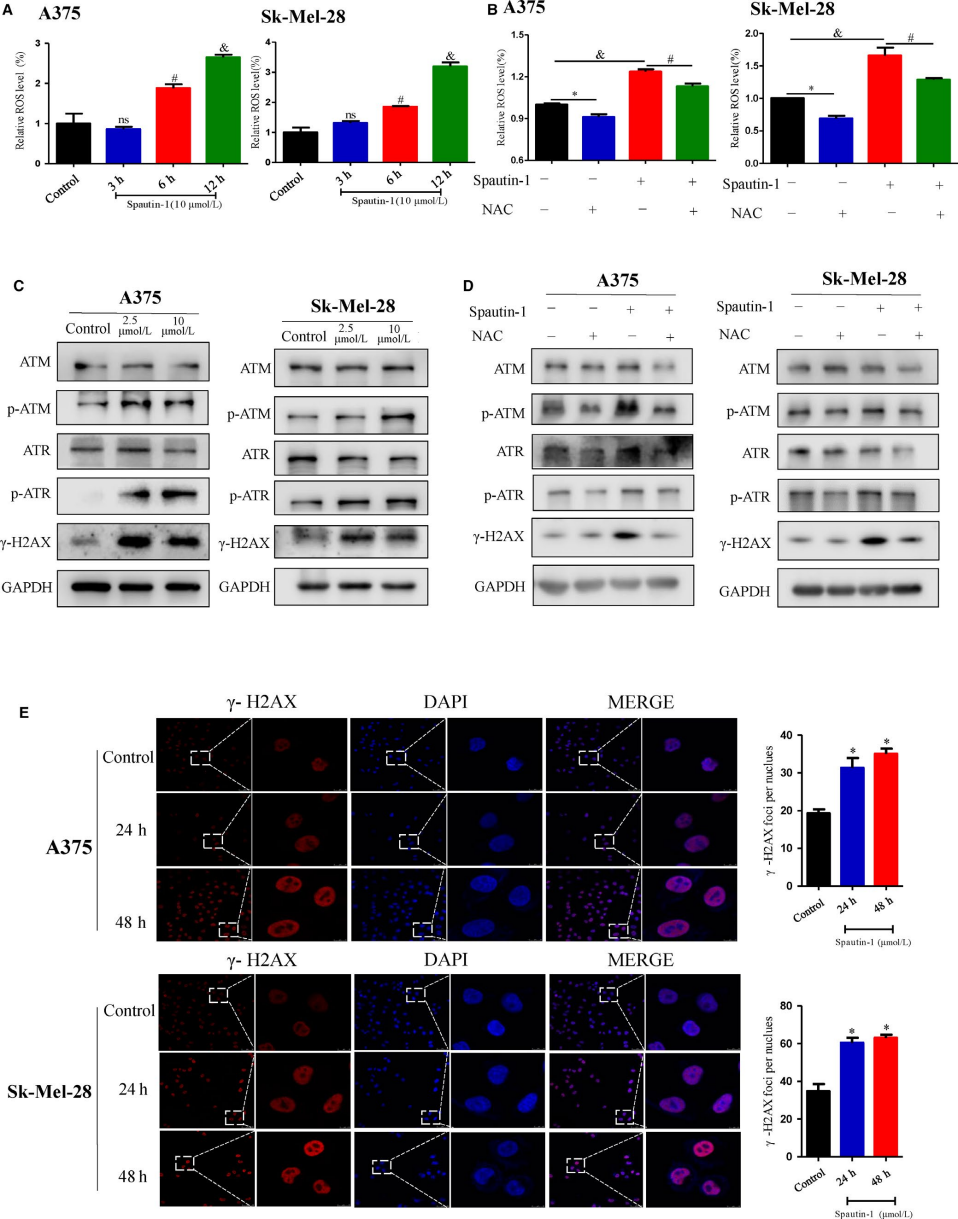
Spautin‐1 caused ROS‐mediated DNA damage in melanoma cells. A, Effects of spautin‐1 on ROS generation in A375 and Sk‐Mel‐28 cells. The cells were treated with 10 μmol/L spautin‐1 for 0, 3, 6 or 12 h. Cells were stained with DCF fluorescence probe and the generation of ROS was measured by flow cytometry. The bar graphs showed the relative ROS levels. (Mean values ± SEM, n = 3) *P < .05. B, A375 and Sk‐Mel‐28 were pre‐treated with or without antioxidant NAC (2 mmol/L) for 1 h and then, exposed to spautin‐1 (10 μmol/L) or control medium for another 6 h. Cells were stained with DCF fluorescence probe and the generation of ROS was measured by flow cytometry. The bar graphs showed the relative ROS levels. (Mean values ± SEM, n = 3) *P < .05. C, Cells were treated with spautin‐1 (control, 2.5 or 10 μmol/L) for 48 h. Effects of spautin‐1 on the expression levels of ATM, p‐ATM, ATR, p‐ATR and γ‐H2AX in A375 and Sk‐Mel‐28 cells were examined by Western blot assay. D, A375 and Sk‐Mel‐28 were pre‐treated with or without antioxidant NAC (2 mmol/L) for 1 h and then exposed to spautin‐1 (10 μmol/L) or control medium for another 6 h. Western blot assay was then implied to detect the indicated protein level. E, (Left panel) Representative images of immunofluorescence staining of γ‐H2AX in A375 and Sk‐Mel‐28 cells treated with 10 μmol/L spautin‐1 for 0‐48 h. (Right panel) Quantitative analysis of γ‐H2AX nucleus foci. (Mean values ± SEM, n = 3) Significant differences were evaluated using a one‐way ANOVA. *P < .05 vs control

Many studies have reported that ROS‐dependent oxidative DNA damage is closely related to inducing G2/M cell cycle arrest and increasing cell apoptosis.^14^, ^15^ The generation of ROS has been proven to play a critical role in the development, metastasis and progression of cancer.^16^ In our research, we hypothesized that spautin‐1 can cause the oxidative DNA damage related to the production of ROS. The induction of intracellular ROS was examined by using DCFDA staining. Interestingly, we observed that intracellular ROS levels were significantly increased in melanoma cell lines after spautin‐1 treatment (Figure 5A) and which could be partly blocked by the antioxidant N‐acetylcysteine (NAC), a known scavenger of ROS (Figure 5B). Furthermore, Western blotting data showed that spautin‐1 treatment for 48 hours effectively increased the expression of γ‐H2AX, p‐ATM and p‐ATR, while there were no clear changes in the expression of total ATM and ATR (Figure 5C). Immunofluorescence staining of γ‐H2AX, a DNA damage marker,^17^ in A375 and SK‐Mel‐28 cells post‐spautin‐1 treatment (10 μmol/L for 0, 24 or 48 hours) showed that accumulation of γ‐H2AX in the nucleus (Figure 5E), indicating that spautin‐1 induced DNA damages. NAC also suppressed the spautin‐1‐induced increase in γ‐H2AX, p‐ATM and p‐ATR activity (Figure 5D), further confirming the indispensable role of ROS accumulation in spautin‐1‐induced activation of DNA damage. Similarly, USP10/USP13 silencing could induce ROS generation and cause DNA damage (Figure S2D‐F). All of the above results indicated that spautin‐1 treatment induces oxidative DNA damage via promoting the accumulation of intracellular ROS.

### 3.5 Spautin‐1 had a synergistic effect with cisplatin in the treatment of melanoma cells by inducing DNA damage in vitro

**Figure 6:**
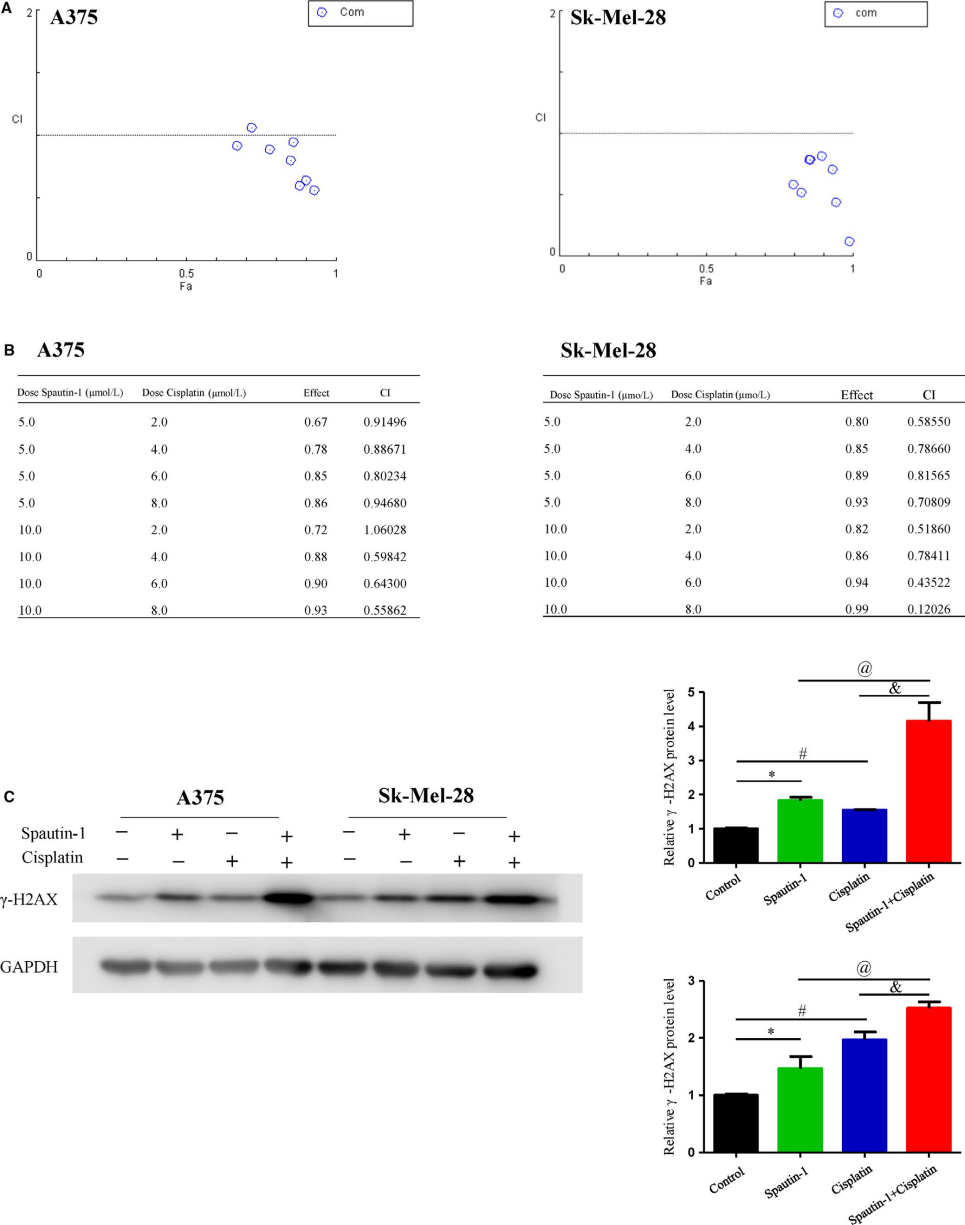
Spautin‐1 had synergistic effect with cisplatin in the treatment of melanoma cells by induction of DNA damage in vitro. A and B, The synergistic effect of spautin‐1 in combination with cisplatin on the growth of A375 and SK‐Mel‐28 cells. Combination index (CI) values were calculated at the drug concentration of spautin‐1 (5 μmol/L) plus cisplatin (2 μmol/L), spautin‐1 (5 μmol/L) plus cisplatin (4 μmol/L), spautin‐1 (5 μmol/L) plus cisplatin (6 μmol/L), spautin‐1 (5 μmol/L) plus cisplatin (8 μmol/L), spautin‐1 (10 μmol/L) plus cisplatin (2 μmol/L), spautin‐1 (2 μmol/L) plus cisplatin (2 μmol/L), spautin‐1 (10 μmol/L) plus cisplatin (4 μmol/L), spautin‐1 (10 μmol/L) plus cisplatin (6 μmol/L) and spautin‐1 (10 μmol/L) plus cisplatin (8 μmol/L) using the Chou‐Talalay method. C, A375 and SK‐Mel‐28 cell lines were treated with DMSO, spautin‐1 (5 μmol/L) and/or cisplatin (4 μmol/L) for 48 h. Western blot analysis of γ‐H2AX protein expression was performed (Left panel). Semi‐quantitative analysis of γ‐H2AX protein expression compared with GAPDH (right panel). (Mean values ± SEM, n = 3) Significant differences were evaluated using a one‐way ANOVA. *P < .05 Sputin‐1 vs control group, ^#^ *P* < .05 cisplatin vs control group, ^&^ *P* < .05 spautin‐1 vs spautin‐1 + cisplatin, ^@^ *P* < .05 cisplatin vs spautin‐1 + cisplatin

To test the clinical relevance and clinical significance of spautin‐1 in the treatment of melanoma, we assessed the synergistic effect of spautin‐1 and several first‐line clinical drugs. Our results showed that there was no synergistic effect of combining spautin‐1 with several first‐line targeted drugs, such as vemurafenib and trametinib (Figure S4C‐F), respectively. However, analysis of the cell viability revealed that a combination of spautin‐1 and cisplatin caused markedly more cell inhibition than spautin‐1 or cisplatin alone within a wide range of drug concentration in A375 and SK‐Mel‐28 cells (Figure S4A,B). A widely accepted quantitative analysis method for drug combination is the Chou‐Talalay method. The properties of drug combination can be quantitatively defined by the value of the combination index (CI), a theorem of Chou‐Talalay. It defines CI = 1 as an additive effect, CI < 1 as a synergistic effect and CI > 1 as an antagonistic effect.^18^ Our data showed that the combination of spautin‐1 and cisplatin at a non‐constant ratio achieved CI < 1 in different melanoma cell lines (Figure 6A), and the particular CI values are shown in Figure 6B. A large number of studies reported that cisplatin attenuated cancer cell growth through inducing DNA damage and apoptosis.^19^, ^20^, ^21^ Previous data suggested that spautin‐1 might inhibit cell proliferation by inducing DNA damage. To further characterize whether the inhibition of cell proliferation in response to spautin‐1 combination with cisplatin was associated with DNA damage, the expression of γ‐H2AX, a recognized marker of DNA damage, was investigated. Western blot analysis showed that after 48 hours, compared with the spautin‐1 or cisplatin alone group, treatment of A375 and SK‐Mel‐28 cells with a combination of spautin‐1 and cisplatin induced a significant increase in the expression of γ‐H2AX (Figure 6C). These results suggest that spautin‐1 treatment could enhance the sensitivity of melanoma cells to cisplatin via heightening the effect of DNA damage.

### 3.6 Spautin‐1 treatment attenuated tumour growth in a cell‐derived xenograft mouse model, and its anti‐tumour effect was further enhanced by cotreatment with cisplatin

**Figure 7:**
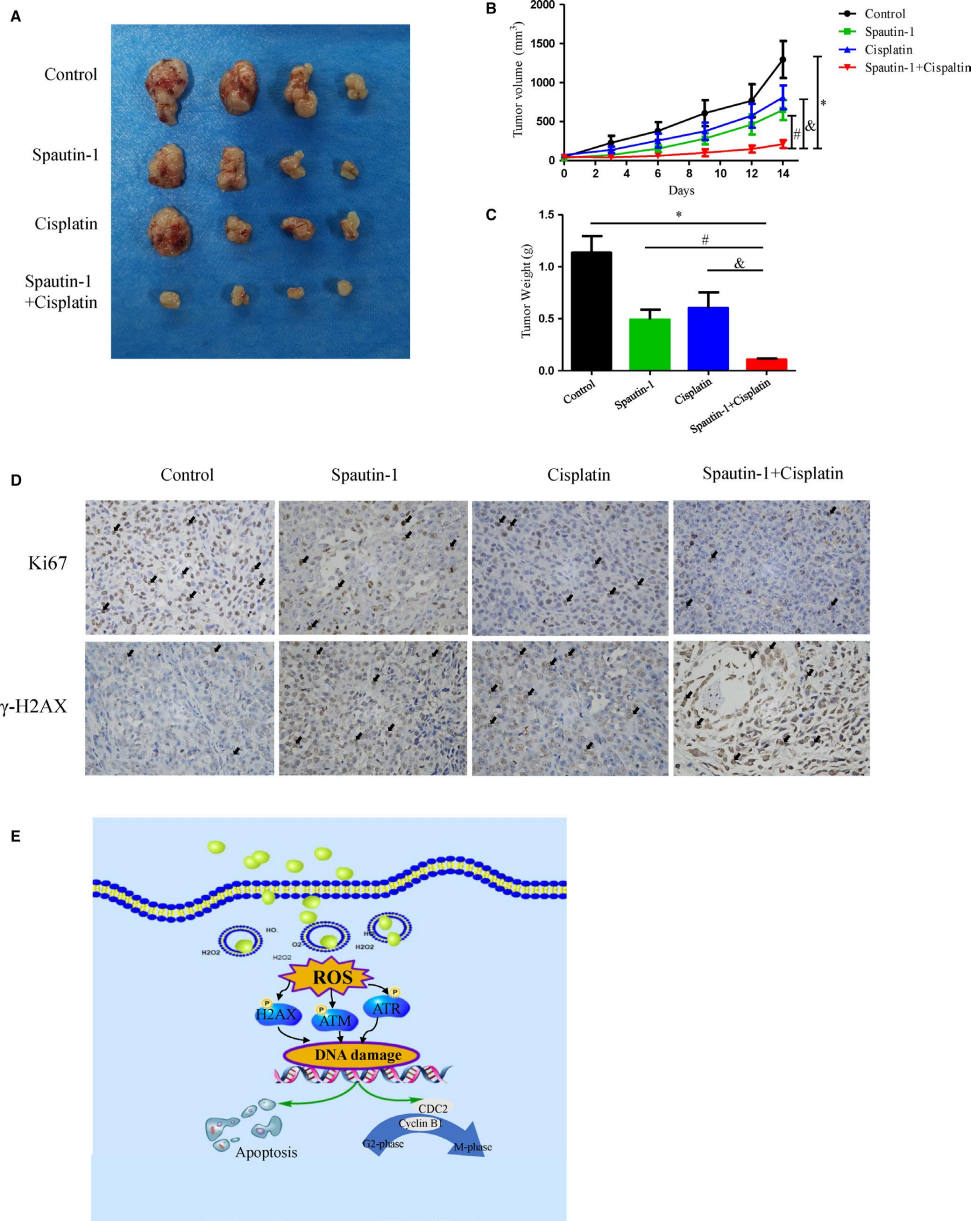
Combination treatment of cisplatin significantly enhances the anti‐tumour effect of spautin‐1 in the treatment of melanoma xenograft tumour models. A‐C, A375 melanoma cells (5 × 10^6^ cells/0.1 mL) were xenografted into nude mice. The mice were randomized for intraperitoneal injection of spautin‐1 (40 mg/kg, once every other day) and/or gavage administration of cisplatin (3 mg/kg, once a week) for 14 days. The bodyweight (C) and tumour growth (B) were measured around twice per week. All the tumours were removed, and the weight was measured for each group at the last day (mean values ± SEM, n = 7). Significant differences were evaluated using a one‐way ANOVA. *P < .05 vs control group, ^#^ *P* < .05 vs spautin‐1 group, ^&^ *P* < .05 vs cisplatin group. D, Immunohistochemistry assay assessed Ki67 (upper) and γ‐H2AX (lower). The representative images are shown. E, Proposed working model of spautin‐1 on melanoma cells

To further investigate the effect of spautin‐1 on malignant melanoma in vivo, a cell‐derived xenograft melanoma model was utilized. The A375 cell line has been adopted to establish subcutaneous xenograft tumour models.^22^ Consistent with our in vitro results, spautin‐1 significantly attenuated tumour growth in vivo and further enhanced the sensitivity of tumours to cisplatin compared to the control group, reflected by smaller tumour sizes and tumour weight (Figure 7A‐C). Meanwhile, the bodyweight of tumour‐bearing mice in all treatment groups had no significant change during this progress, ruling out the toxicity of spautin‐1 in vivo (Figure S4B). Immunohistochemical staining of the tumour tissues showed decreased expression of Ki‐67, a well‐accepted proliferation marker, in the spautin‐1‐treated group compared with the control group, with further decreased expression in the combination group compared with the single‐drug group (Figure 7D). In addition, histopathological findings of the tumours showed that the expression of γ‐H2AX in the combined drug group was significantly higher than in the single‐drug group (Figure 7D), which was consistent with previous results. Therefore, these results suggested that spautin‐1 alone or combined with cisplatin could significantly inhibit the proliferation of malignant melanoma in vivo.

## 4 DISCUSSION

In human tumours, DUBs play an important role in the occurrence and development of tumours. Therefore, DUBs represent novel candidates for target‐directed drugs to inhibit the progression of tumours. DUBs have become a trend in the field of drug development.^10^, ^23^ Recent studies have shown that USP10 and USP13 function as tumour promoters in human cancer by stabilizing multiple molecules.^24^ However, the functions of USP10/13 in malignant melanoma are still unknown. In the present study, we discovered the high expression levels of USP10 and USP13 in malignant melanoma tissues and explored the function and underlying mechanisms of the anti‐melanoma effects of the USP10/USP13 inhibitor spautin‐1 through inducing the ROS‐mediated DNA damage. Furthermore, we found that spautin‐1 remarkably suppressed the proliferation of melanoma cells alone and had a synergistic effect with cisplatin in the treatment of melanoma both in vivo and in vitro.

Spautin‐1, a USP10/USP13 inhibitor, was also reported to be an autophagy inhibitor by enhancing the degradation of beclin‐1, a protein required for the initiation of autophagy.^12^ It was reported that loss of beclin‐1 could lead to DNA damage.^25^ Previous study showed that spautin‐1 inhibits cell growth and angiogenesis through inactivating the PI3K/AKT pathway in chronic myeloid leukaemia.^26^ In particular, it can suppress the growth of ovarian cancer and lung cancer by regulating MCL1 stability.^24^ However, its role in malignant melanoma has not been appreciated thus far. In this study, we revealed that spautin‐1 is a novel and a safe approach to inhibiting malignant melanoma proliferation.

Mechanistically, by utilizing RNA‐sequencing coupled with qRT‐PCR, we identified enriched pathways and genes involved in the potential molecular mechanisms of the anti‐proliferative role of spautin‐1 in melanoma. As such GADD45A and GADD45B were significantly increased after spautin‐1 treatment. These two proteins were targets of DNA damage, and they played critical roles in apoptosis and G2/M checkpoint in response to DNA damage.^27^, ^28^, ^29^ In addition, BTG2, a cell cycle control gene, initiated the G2/M phase arrest and inhibited cell growth and was induced in response to DNA damage mediated by p53‐dependent mechanisms.^30^, ^31^ Reactive oxygen species (ROS), such as H_2_O_2_, superoxide anion ($O_{2}^{-}$) and hypochlorous acid (HOCl),^32^ were recognized as a mediator of DNA damage.^33^ The accumulation of high levels of ROS may cause further DNA damage and then induce cell death.^34^, ^35^, ^36^ In this study, we found that spautin‐1 significantly induced rapid ROS generation. Furthermore, we revealed that spautin‐1 caused DNA damage. Previous study reported that the overload of mitochondria ROS resulted in the reduction of mitochondrial membrane potential (MMP) and subsequently caused the cellular apoptosis.^37^, ^38^, ^39^ We assessed the changes of the MMP in the spautin‐1‐treated cells with a JC‐1 probe.^40^ The cells showed a sharp MMP decline after being treated with spautin‐1 (Figure S5). The cytometry results showed that spautin‐1 treatment significantly induced melanoma cell apoptosis (Figure 4B). We further utilized Western blotting to reveal the molecular mechanism of spautin‐1‐mediated proapoptotic effect in melanoma. PARP, a well‐known DNA strand break‐binding enzyme, is cleaved and activated and then causes apoptosis.^41^ BCL‐2, as an apoptosis regulator, can block the apoptotic death of cancer cell. BAX belongs to the BCL2 protein family, functions as an apoptotic activator.^42^ Our results demonstrated that spautin‐1‐induced cellular apoptosis by up‐regulating cleaved‐PARP and BAX and down‐regulating of BCL‐2 (Figure 4D).

To further test the clinical relevance of spautin‐1 in the treatment of melanoma, we screened the synergistic effect of spautin‐1 with other targeted drugs, including vemurafenib and trametinib, and chemotherapeutic drugs, such as cisplatin. Our results showed that spautin‐1 and cisplatin combination treatment showed potent synergistic effects in melanoma cell lines in vitro and in vivo. Cisplatin, as a well‐known chemotherapeutic drug, plays an anti‐tumour role through a variety of mechanisms, the most important of which is to activate the DNA damage response and induce apoptosis.^43^, ^44^ The cisplatin benefits the overall survival (OS) and relapse‐free survival (RFS) of advanced melanoma and metastatic melanoma patients.^45^ However, single‐agent chemotherapy regimens have produced low response rates, and the median response duration is only 4‐5 months.^46^, ^47^ According to reported studies, cisplatin is used for advanced melanoma in combination with other chemotherapeutic drugs or after targeted drugs.^20^, ^48^ Our results showed that spautin‐1 inhibited melanoma cell proliferation by ROS‐induced DNA damage, and spautin‐1 had a synergistic effect with cisplatin, but not vemurafenib or trametinib. Vemurafenib (selective BRAF inhibitors) and trametinib (Inhibitors of the downstream MAP kinase MEK) were targeted inhibitors utilized for the treatment of melanoma. Previous studies reported that the molecular mechanism of vemurafenib‐ and trametinib‐mediated anti‐tumour effects was inhibition of MAPK pathway.^49^, ^50^ It was reported that elevation of ROS activated MAPK‐related pathway.^51^, ^52^, ^53^ Therefore we speculated that spautin‐1 had no synergistic effect with vemurafenib or trametinib may be due to the opposite effect in terms of regulating MAPK pathways in the treatment of melanoma. Taking together, spautin‐1 may be not only a potential therapeutic approach for melanoma cells but also a sensitizer for cells that are resistant to conventional chemotherapeutic agents, especially cisplatin.

Taken together, our study revealed that spautin‐1 induced G2/M cell cycle arrest and cell apoptosis via up‐regulation of ROS‐mediated DNA damage and had a synergistic effect with cisplatin (Figure 7E), which indicated that spautin‐1 mediated beneficial effects in melanoma and identified a potential therapeutic strategy in the treatment of melanoma patients.

## CONFLICT OF INTEREST

The authors declare that they have no competing interests.

## AUTHORS’ CONTRIBUTIONS

HL and XC developed the hypothesis, designed the experiments, and revised the manuscript. JG and JLZ conducted experiments and wrote the main manuscript. LL, NL and MQ assisted in experiments and manuscript writing. SZ, JS, JL and CP performed the statistical analyses.

## ETHICS APPROVAL AND CONSENT TO PARTICIPATE

The animal protocol was approved by the Ethics Committee of Xiangya Hospital (Central South University, China).

## Supporting information

Supplementary Material

## Data Availability

The data sets used and/or analysed during the current study are available from the corresponding author on reasonable request.
