# Supplementary material for: Potent USP10/13 antagonist spautin‐1 suppresses melanoma growth via ROS‐mediated DNA damage and exhibits synergy with cisplatin
Source: J Cell Mol Med. 2020 Mar 4;24(7):4324–40. doi: 10.1111/jcmm.15093 (PMC7171391; doi:10.1111/jcmm.15093)
Supplement: Supplementary file 1 — Supplementary Material [file JCMM-24-4324-s001.docx]

**
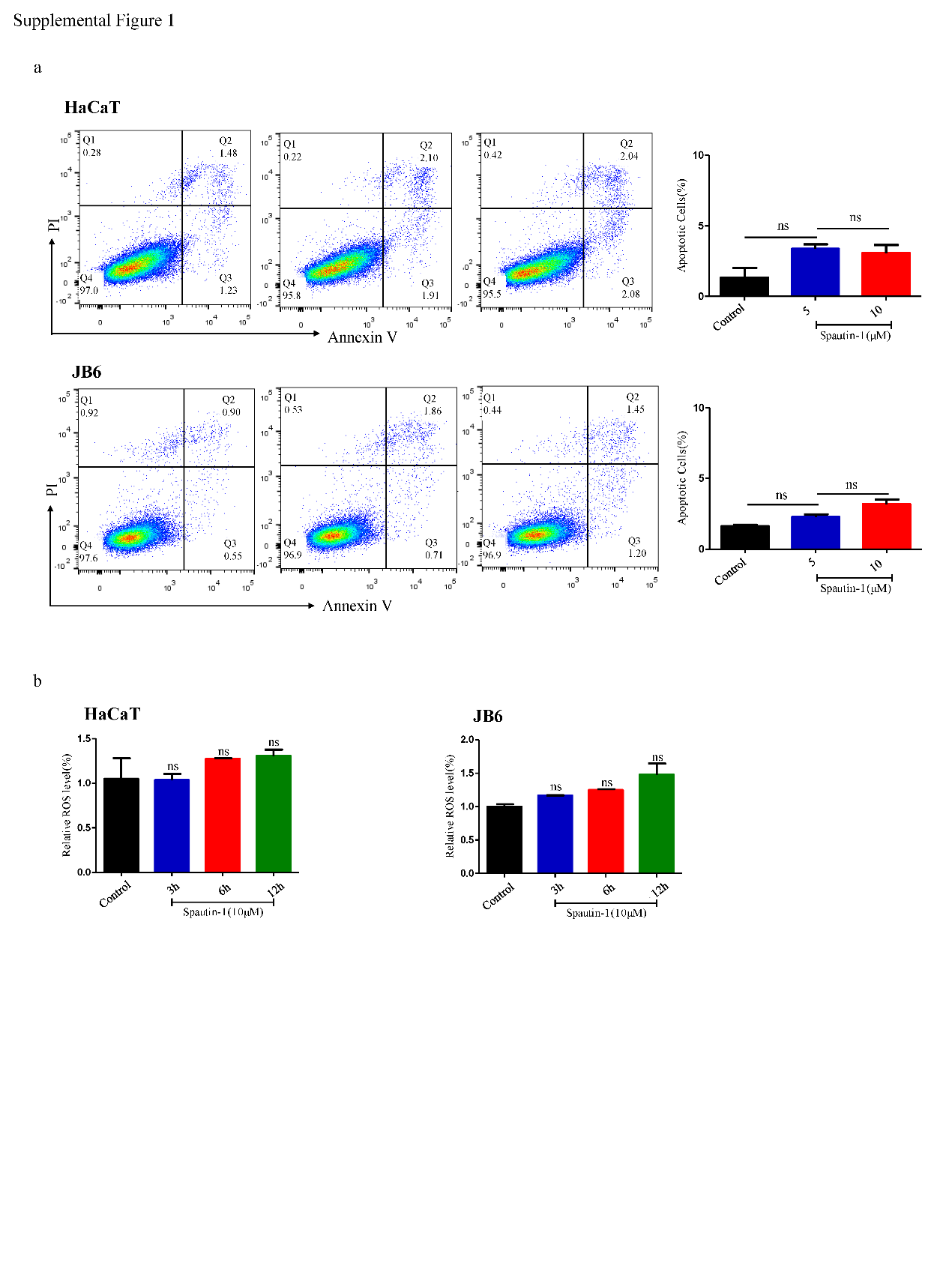
**

**Supplemental Figure 1**

**The effect of Spautin-1 on the control cells**

1. (Left) Representative data of apoptosis of Spautin-1-treated cells. HaCat and JB6 cells were disposed with different dosages of Spautin-1 for 48 h and then cells were stained with annexin V and PI to measure the percentage of death cells. (Right) The bar graphs showed the percentage of death cells. (Mean values ± SEM, n = 3) Significant differences were evaluated using a one-way ANOVA. ns P>=0.05 vs control group. (b) Cells were treated with 10μM Spautin-1 for 0 h, 3 h, 6 h or 12h and then stained with DCF fluorescence probe and the generation of ROS was measured by flow cytometry. The bar graphs showed the relative ROS levels. (Mean values ± SEM, n = 3) Significant differences were evaluated using a one-way ANOVA. ns P>=0.05 vs control group.

**
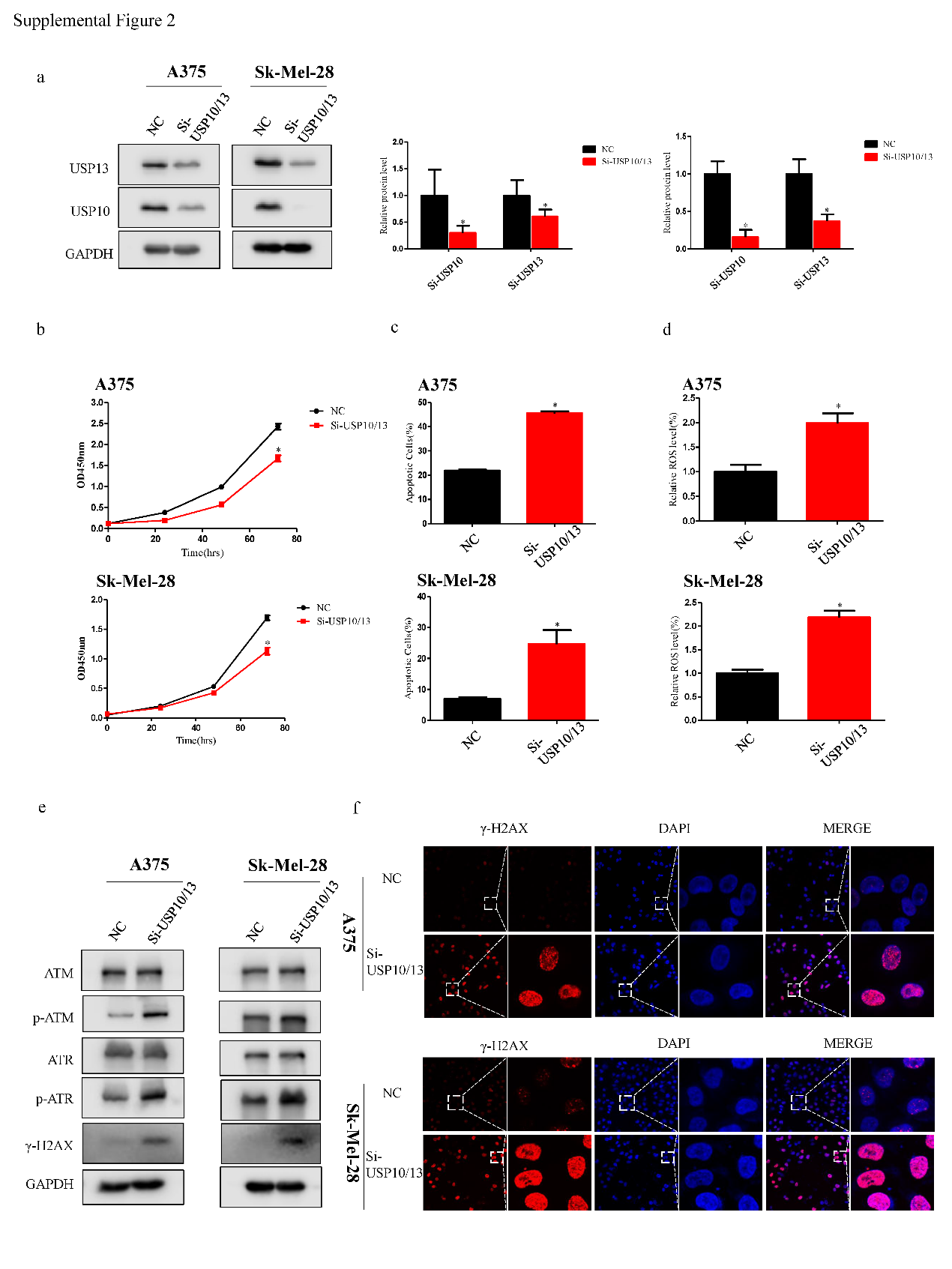
**

**Supplemental Figure 2**

**USP10 and USP13 regulate tumor proliferation via ROS-mediated DNA damage pathways**

1. USP10/13-knockdown cells were generated by using different sequences as described in Materials and methods. The expression of USP10 and USP13 was assessed by western blotting as indicated (Left panel). Significant differences were evaluated using Student’s t-test. * P<0.05 vs Negative Control (NC). (b) Knockdown of USP10 and USP13 attenuated the proliferation of A375 and Sk-Mel-28 cells. USP10/USP13-knockdown cells were generated by using separated sequences as described in Materials and methods. The USP10/USP13 silenced cells were seeded into 96-well plates, and proliferation was assessed by MTS as described in the Materials and Methods and analyzed by GraphPad Prism software (Mean values ± SEM, n = 6). (c) Cell apoptosis was evaluated by flow cytometry and analyzed by GraphPad Prism software. Significant differences were evaluated using Student’s t-test. * P<0.05 vs Negative Control (NC). (d) USP10/USP13-silenced cells induced more generation of ROS than the control cells. The NC and USP10/USP13 knockdown cells were stained with DCF fluorescence probe and then the generation of ROS was measured by flow cytometry. The file was analyzed by GraphPad Prism software. Significant differences were evaluated using Student’s t-test. * P<0.05 vs NC. (e) Knockdown of USP10 and USP13 induced activation of DNA damage. Effects of USP10/USP13 silence on the expression levels of ATM, p-ATM, ATR, p-ATR and γ-H2AX in A375 and Sk-Mel-28 cells were examined by western blot assay. (f) Representative images of immunofluorescence staining of γ-H2AX in USP10/USP13-knockdown A375 and Sk-Mel-28 cells.

**
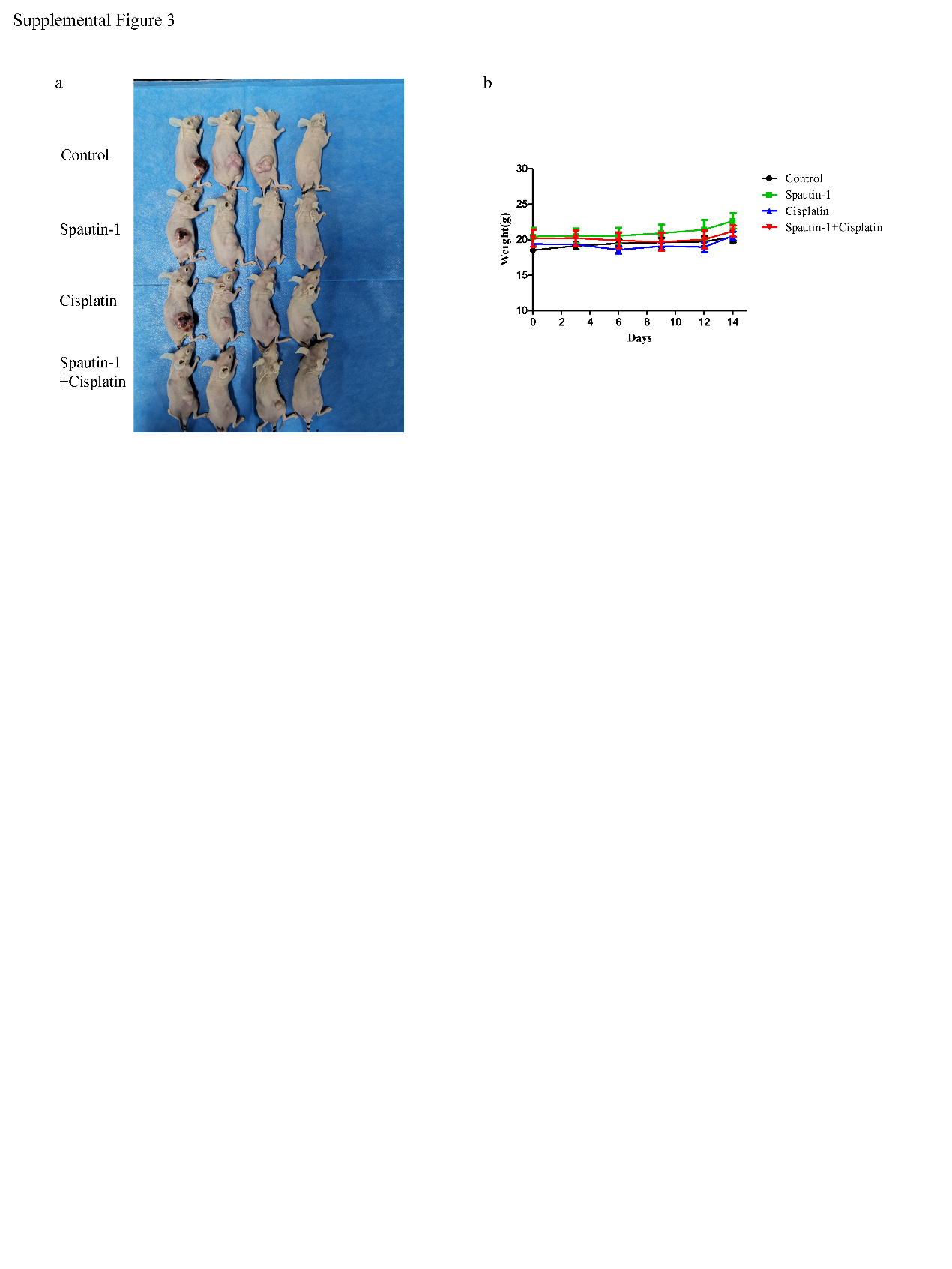
**

**Supplemental Figure 3**

**Spautin-1 contributes to treatment of melanoma xenograft tumor models and have synergistic effect with cisplatin**

(a) A375 melanoma cells (5 × 106 cells/0.1 mL) were xenografted into nude mice. The mice were randomized for intraperitoneal injection of Spautin-1 (40mg/kg, once every other day) and/or gavage administration of Cisplatin (3mg/kg, once a week) for 14 days. The overview of nude mice was showed. (b) The body weight of mice was measured twice per week. (Mean values ± SEM, n = 7).

**
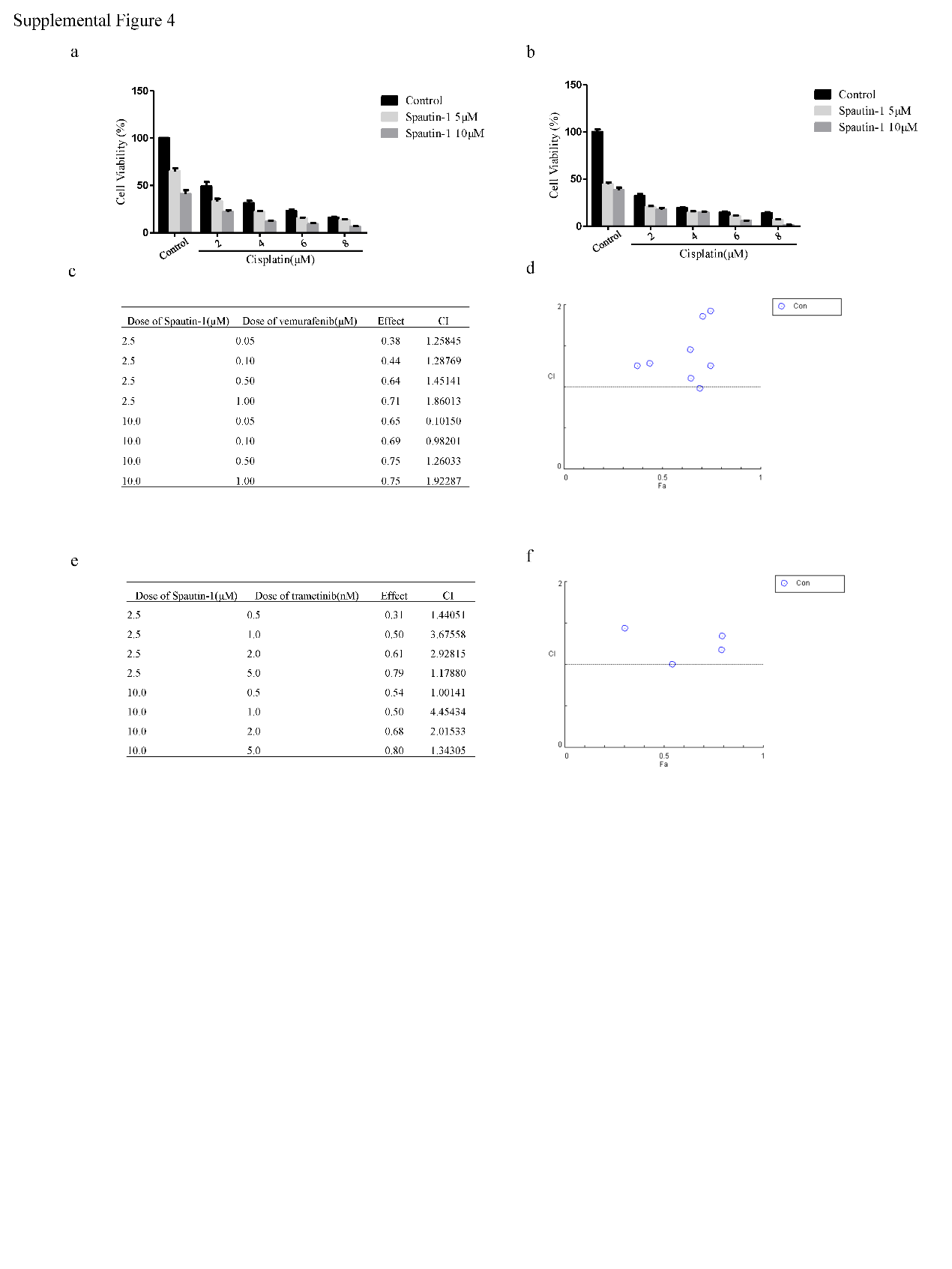
**

**Supplemental Figure 4**

**The interaction of Spautin-1 and Cisplatin/ Vemurafenib/ Trametinib**

(a&b) A375 and Sk-Mel-28 cells were treated with Spautin-1(0μM, 5μM, 10μM) and

Cisplatin (0μM, 2μM, 4μM, 6μM, 8μM) concentrations at 72h. The cell viability was measured by MTS assay as described in the Materials and Methods. (Mean values ± SEM, n = 6) (c&d) The antagonistic effect of Spautin-1 in combination with Vemurafenib on the growth of SK-Mel-28 cells. Combination index (CI) values were calculated at the drug concentration of Spautin-1 (2.5μM) plus Vemurafenib (0.05μM), Spautin-1 (2.5μM) plus Vemurafenib (0.1μM), Spautin-1 (2.5μM) plus Vemurafenib (0.5μM), Spautin-1 (2.5μM) plus Vemurafenib (1μM), Spautin-1 (10μM) Vemurafenib (0.05μM), Spautin-1 (2μM) plus Vemurafenib (0.1μM), Spautin-1 (10μM) plus Vemurafenib (0.5μM) and Spautin-1 plus Vemurafenib (1μM)using the Chou-Talalay method. (E&F) The antagonistic effect of Spautin-1 in combination with Trametinib on the growth of SK-Mel-28 cells. Combination index (CI) values were calculated at the drug concentration of Spautin-1 (2.5μM) plus Trametinib (0.05nM), Spautin-1 (2.5μM) plus Trametinib (1nM), Spautin-1 (2.5μM) plus Trametinib (2nM), Spautin-1 (2.5μM) plus Trametinib (5nM), Spautin-1 (10μM) plus Trametinib (0.05nM), Spautin-1 (2μM) plus Trametinib (1nM), Spautin-1 (10μM) plus Trametinib (2nM) and Spautin-1 plus Trametinib (5nM) using the Chou-Talalay method.


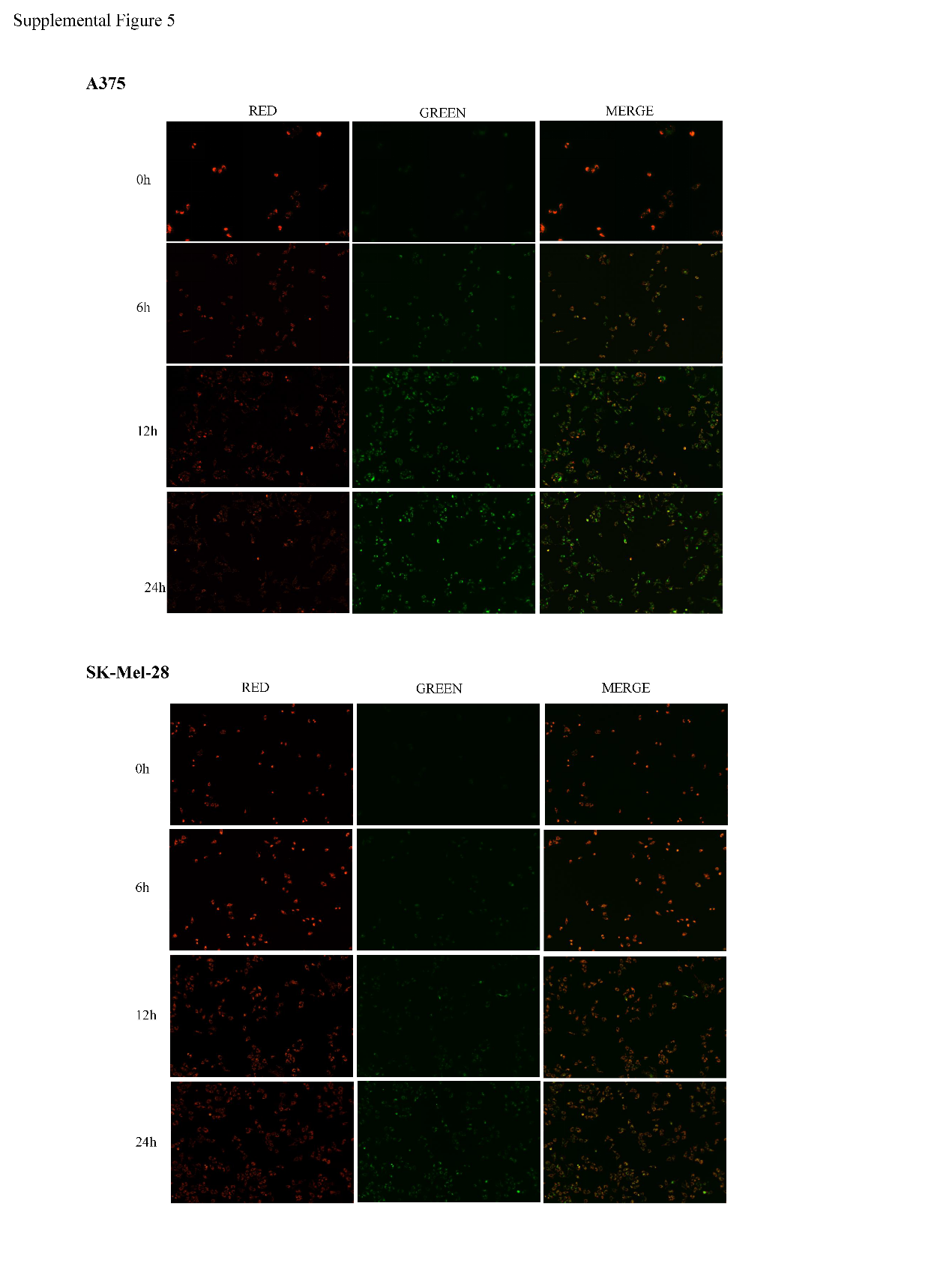


**Supplemental Figure 5**

 A375 and Sk-Mel-28 cells subjected to 10μM dose for different time intervals (0, 6,12 or 24 h) were stained by JC-1. Change of MMP was detected by [fluorescence microscopy](https://www.sciencedirect.com/topics/medicine-and-dentistry/fluorescence-microscopy). The transition of red fluorescence to green fluorescence means decline of MMP.

**Supplemental table**

**The primers used in the PCR reaction and annealing**

**
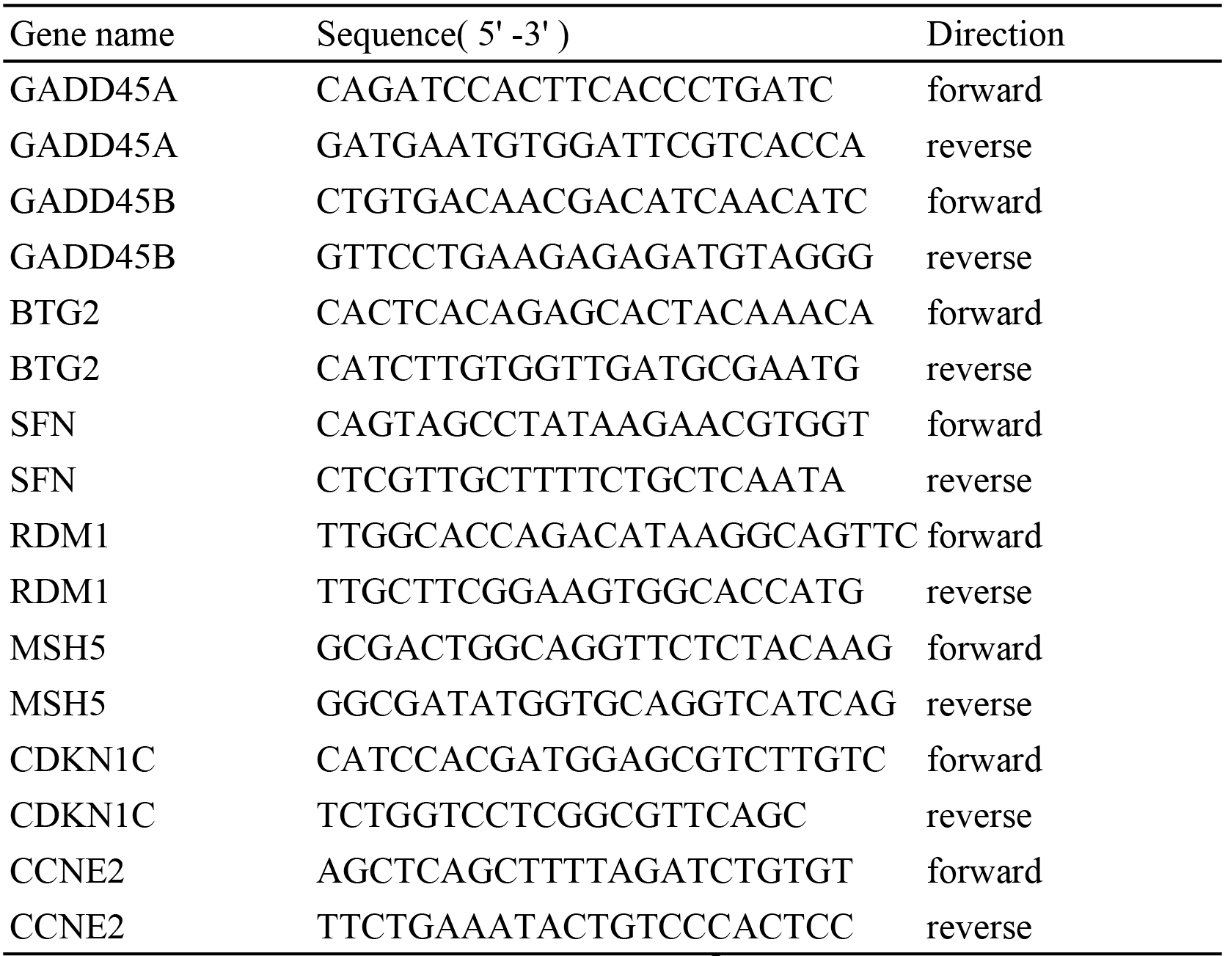
**
